# Reflecting on the Reasons Pros and Cons Coercive Measures for Patients in Psychiatric and Somatic Care: The Role of Clinical Ethics Consultation. A Pilot Study

**DOI:** 10.3389/fpsyt.2019.00441

**Published:** 2019-06-20

**Authors:** Elena Montaguti, Jan Schürmann, Charlotte Wetterauer, Mario Picozzi, Stella Reiter-Theil

**Affiliations:** ^1^Biotechnology and Life Sciences Department, University of Insubria, Varese, Italy; ^2^Department of Clinical Ethics, University Hospital Basel (USB), Basel, Switzerland; ^3^Department of Clinical Ethics, University Psychiatric Hospital (UPK), Basel, Switzerland; ^4^Department of Clinical Ethics, University Hospital Basel (USB) and University Psychiatric Hospital (UPK), University of Basel, Basel, Switzerland

**Keywords:** coercion, ethics consultation, case series, law, guidelines, psychiatry, somatic care

## Abstract

**Background and aim:** Coercive measures in patient care have come under criticism leading to implement guidelines dedicated to the reduction of coercion. This development of bringing to light clinical ethics support is hoped to serve as a means of building up awareness and potentially reducing the use of coercion. This study explores the specific features of ethics consultation (EC) while dealing with coercion.

**Material and method:** Basel EC documentation presents insight to all persons involved with a case. The EC database of two Basel university hospitals was developed on the grounds of systematic screening and categorization by two reviewers. One hundred fully documented EC cases databased from 2013 to 2016 were screened for the discussion of coercive measures (somatic hospital and psychiatry: 50% cases).

**Results:** Twenty-four out of 100 EC cases addressed coercion in relation to a clinically relevant question, such as compulsory treatment (70.8%), involuntary committal (50%), or restricting liberty (16.6%). Only 58.3% of EC requests mentioned coercion as an ethical issue prior to the meeting. In no case was patient decisional capacity given, capacity was impaired (43.5%), not given (33.3%), or unclear (21.7%; one not available).

**Discussion:** As clinical staff appears sensitive to perceiving ethical uncertainty or conflict, but less prepared to articulate ethical concern, EC meetings serve to “diagnose” and “solve” the ethical focus of the problem(s) presented in EC. Patient decisional incapacity proved to be an important part of reasoning, when discussing the principle of harm prevention. While professional judgment of capacity remains unsystematic, rationality or even ethicality of decision making will be hampered. The documented EC cases show a variety of decisions about whether or not coercion was actually applied. Ethical reasoning on the competing options seemed to be instrumental for an unprejudiced decision complying with the normative framework and for building a robust consensus.

**Conclusions:** The recommendation is whether EC should be used as a standard practice whenever coercion is an issue—ideally before coercion is applied, or otherwise. Moreover, more efforts should be made toward early and professional assessment of patient capacity and advance care counseling including the offer of advance directives.

## Introduction

In constitutional states such as Switzerland, the use of coercion against persons requires explicit legal legitimation (see below); moreover, within this legal framework, ethical justification is regarded to be mandatory. Medicine and healthcare workers are, therefore, obliged to consistently justify any limitation of their patient’s personal freedom within reason, specifically to prevent harm to the patient or others. Some of the questions regarding the use of coercion are, e.g.: How to appropriately manage an inpatient violating the house rules and refusing long-term medication? Is involuntary hospitalization of an incompetent patient with aggressive and self-harming behavior justified? Examples such as these illustrate that the lack of insight and cooperation as well as aggressive patient behavior are central issues that indicate not only a psychiatric context. Critically ill patients in somatic care may also trigger discussion on coercive measures. Coercion may concern treatment, diagnostic measures, patient location, accommodation, and social environment. It may also affect the therapeutic alliance between patient and therapist and, thus, cause problems for the involved healthcare professional (1–3). Quantitative data on coercive measures applied in patient care are “hard to compare, since coercive measures are rarely systematically recorded, nor calculated and analyzed or expressed in a consistent way”; however, “studies in Europe and the US indicate that 10 to 30% of all admitted patients are exposed to seclusion, restraint, or forced medication in acute psychiatric wards” as Janssen et al. report (p. 430) (4). Only since 2015, Swiss National Association for Quality Development in Hospitals and Clinics (ANQ) assessment of the exertion of coercive measures has been systematically carried out throughout Switzerland. Previously, Lay et al. (5) collected quantitative data. However, as stated by the authors “for ethical and clinical reasons it is therefore indispensable to scrutinise the use of coercive measures and to investigate the conditions under which these procedures are justified and effective” (p. 250) (5). Studies focusing on the ethics of coercion in healthcare are still rare, especially studies shedding light on ethics consultation (EC); the inclusion of a qualitative research approach befitting exploration of new terrain is required. Moral distress or ethical challenges experienced by staff facing coercive measures have been investigated in recent papers from the Netherlands and Norway (1, 2). Norvoll et al. evaluated seven telephone interviews with “key informants” in Norwegian mental healthcare institutions who had not, as of yet, used ethics support; he then came to the encouraging conclusion that an “explicit use of formal clinical ethics support” might help to “opening up a moral space in health care facilities” (6). More specifically, Syse’s (et al.) report on the Norwegian clinical ethics committees showed that from 144 mental health and addiction treatment cases, 23 addressed “dilemmas related to coercion (formal and informal)” (p. 83) (2016). She concluded: “Given the field’s ethical weight of seriously ill and vulnerable patients, it seems reasonable to suggest that focus on mental health care and addiction in ethics committees should be strengthened (p. 86) (7).

Coercive measures have increasingly been put to the test bench in various countries—a movement that has led to the implementation of policies dedicated to the reduction of any form of coercion in medicine, e.g. in Germany (8), Norway (9), or the Netherlands (10). This development has been associated with the effort to promote clinical ethics support (CES) that is hoped will serve as a means of increasing awareness and potentially reducing the use of coercion (11). Completing the picture, it must, however, be acknowledged that coercive measures may, where justified, even be considered necessary, e.g. to restore a dehydrated patient’s decisional capacity by infusion of liquid and medication, or to prevent self-destructive behavior in a patient occurring as a symptom of a treatable and, therefore, reversible pathological condition. Typically, clinical ethics has to address the coexistence of contradictory obligations: here, the duty to respect and the duty to protect is a matter of *phronesis* to distinguish between situations requiring one or the other priority, and CES is attributed in the potential to improve the process and outcome of such ethical deliberation. Any presupposition depreciating coercion in general as “unethical” would be simplistic, neglecting the needs for ethical and practical orientation originating in situations of urgency and emergency where competing values have to be weighed.

Taking the difficulty of this distinction into account, a national guideline on coercive measures was issued by the Swiss Academy of Medical Sciences in 11/2015 (12). At the same time, a systematic case series of 100 EC cases in one major Swiss medical center (Basel) revealed—firstly—the striking result that coercion ranged indeed among the top themes of EC, referring to coercion as the use “of pressure, force or covert action to control the movement, treatment or behavior of a patient against his/her will” (p. 62) (13). To further investigate, the study presented here was initiated to offer a follow-up analysis of those cases, of the same sample, that included the discussion of coercion. Against the background of current literature, the presented case series analysis on 100 ECs addressing the ethical reasoning on coercive measures is new, both in content as well as in design.

### Research Questions

How frequently are coercive measures discussed in this sample of 100 EC cases? Which types of coercion are considered or acknowledged? What are the reasons pros and cons using coercion found in the EC documentation? How is the EC service being evaluated by the requestors according to outcome criteria such as consensus, implementation of results, and helpfulness?

## Legal Framework and Guidelines

Medical coercive measures include mainly: measures restricting liberty, compulsory treatment, and involuntary committal/detention of persons admitted voluntarily. The right to self-determination and the right to liberty are affected by coercive measures, both of which are guaranteed by the European Convention of Human Rights (especially Articles 5 and 8) as well as by Swiss National Law (Article 10 Swiss Federal Constitution). Therefore, restriction of these rights requires special legal legitimation. Coercive measures are only permissible as an exception and may be considered as *ultima ratio* [see Ref. (14)].

Legal requirements provide a framework for answering questions arising in the area of conflict between respect for autonomy, beneficence, and non-maleficence. In Switzerland, the Swiss Child and Adult Protection Law was established in 2013. The Swiss Child and Adult Protection Law helped to harmonize the national legal structure with regard to medical coercive measures. Few specific issues remain within the cantonal responsibility and can lead to cantonal differences like the aftercare for patients after involuntary committal in Article 437 Swiss Civil Code (SCC).

In patients lacking capacity, coercive measures may become unavoidable if, in spite of vigorous efforts, an imminent risk to welfare cannot be averted with the agreement of the person concerned. In patients with decisional capacity, coercive measures are principally not permissible according to Swiss law. They can only be applied in connection with an involuntary committal, in the execution of penal measures, under the Epidemics Act, or possibly on the basis of cantonal regulations—e.g. the possibility for compulsory treatment in a context of somatic disorders, § 26 of the Canton Zurich Patients Act, LS 813.

Involuntary committal is when a person is involuntarily admitted to an appropriate institution for treatment and care. A prerequisite for the ordering of involuntary committal according to Article 426 SCC is the existence of a debilitating condition (mental disorder or disability, or severe neglect) necessitating treatment or care that cannot be provided by other means than through involuntary committal to an appropriate institution. Incapacity is not a requirement for the ordering of involuntary committal (see above). While involuntary committal is always a coercive measure, it does not necessarily mean that the person concerned may be subjected to compulsory drug treatment (12). Compulsory treatment may only be undertaken in cases where the patient lacks capacity, intrusive alternative measures are available no less, and treatment has been ordered in writing by the chief physician (Article 434 SCC) or is required in an emergency (Article 435 SCC). In relation to a treatment decision, a person either has or lacks capacity (12). In individual cases, it may be very difficult to determine whether or not a person has capacity. According to Article 16 SCC, capacity is generally presumed to be present. Complementing the legal provisions, the national Swiss Academy of Medical Sciences (SAMS) guidelines on coercive measures (12) explain how to apply coercive measures. The intention is to raise awareness that any coercive measure, even if it complies with all procedural requirements, constitutes a serious infringement on constitutionally enshrined personal rights and, therefore, requires ethical justification.

## Approach of Clinical Ethics Support

The University Hospital Basel (USB), somatic medicine of adult patients, and the Psychiatric University Hospital Basel (UPK) are autonomous institutions sharing one Department of Clinical Ethics, providing a CES service and collaborating with two different advisory boards. Overall, the EC service is focused “on demand” from all healthcare professionals within the respective institution, but it is also open to patients, their family, or legal substitute decision-makers (15). As most of the EC meetings are triggered by clinical staff, their goals refer to reconsidering or optimizing the respective treatment plan. Whenever goals are articulated, apart from content-wise-defined requests of healthcare professionals, a propensity is to obtain assistance or guidance in finding one’s way toward an ethically sound procedure and conclusion on open or controversial questions. Patients or their relatives prefer to formulate their own wishes for being heard and advised on their options or concerns and to receive help in making themselves better understood by their clinical vis-à-vis. Building an explicit consensus is an important aim of EC that is evaluated regularly and thoroughly. Any treatment (or other intervention, e.g. placement) decision is not permitted to be delegated to an “ethical” authority. Relatively, the responsibility for treatment decisions remains where it has always been, i.e. in the agreement between the physician in charge and the patient or substitute decision-maker relying on shared decision making. (Some decisions may be taken by nurses or social workers and the patient party). The precise locus of the decision making responsibility may be clarified by means of an EC. The Basel approach and ethical framework (16) refers to the four principles of biomedical ethics (17), the concept of a systematic change of perspectives [e.g. Refs. (18, 19)], an escalating repertoire of ways of how to deal with the normative dimension (20), and elements of discourse ethics (21). The role of EC and the chair is, according to the Basel approach following the concept of ethics facilitation (22), not directive: The leading ethicist does not make judgments or decisions about using coercion or not. Rather, following an escalating model, the ethics consultant’s repertoire covers a wide range of activities used for a clear and intersubjective problem analysis and consensus building on problem solution (20). This is associated with honoring the experiences reported and views shared by the clinical staff, patient representatives, or other stakeholders as well as the normative-ethical framework of laws and guidelines. As a result, every EC works as a single-case assessment with an explicit process of agreement on the further procedure.

## Material and Method

This article provides an in-depth analysis of the published Basel EC case series (13). The case series uses a database developed on the grounds of systematic screening and categorization of EC documentations. Basel EC documentation (minutes) is highly structured in sections, mostly co-authored by a junior clinical ethicist and the EC chair acknowledging comments from the participants. It includes a front page containing the explicit demand and initial questions, the meeting’s participants, any preliminary options (for problem solution), and the final ethical and practical conclusions qualified by information on the consensus. This is followed by an overview of (medical) facts and information, especially about the patient’s situation. Next, ethical and legal aspects are formulated explicitly as well as any observations about the course of reasoning. Minutes close with the rehearsal of the ethical conclusions (page 1) complemented by articulating further steps, e.g. how the patient should be informed and by whom, open questions or obligations regarding follow-up. Usually, the minutes cover five or more pages. The single front page serves for quick orientation, while the full document shall enhance transparency for nonparticipants on the reasoning and conclusions considered useful if doubts or controversy arise [according to Ref. (23)]. Attached is a feedback form to be filled in by the requesting party. This form has standard and open questions that serve as a brief evaluation for the requesting party after the meeting (return rate for this sample: 54.2%).

For this study, 100 EC cases from February 2012 to November 2015 of the (more comprehensive) EC database of the Basel University Hospitals were screened for coercion as the main ethical issue, leading to a sample of 27 EC cases. Three of these cases were excluded from analysis, as they dealt with issues of organizational ethics of coercion (rather than clinical) referring to, at the time: The salient process of opening the doors of a psychiatric ward (24). No children were included. The documentation of the resulting final sample of 24 EC cases was screened by two reviewers using content analysis. The following predefined categories were used: type of coercive measures addressed, coercive measures agreed on in the EC conclusion, previously existing involuntary committal, issue of coercion stated in the EC request, reasons pros and cons coercive measures. The reasons pros and cons coercion were further analyzed according to the four principle approaches: respect for autonomy, beneficence, non-maleficence,^1^ and justice (17). Further categories from the first case series regarding demographic and clinical characteristics of the patients involved, such as sex, age, disease, decisional capacity, or prognosis, were included in the analysis [cf. Ref. (13)]. Data were statistically analyzed using IBM SPSS Statistics 25. USB-EC and UPK-EC were analyzed comparatively due to their different core competencies in patient care including management of coercive measures.

## Results

Twenty-four EC cases mainly address ethical issues related to coercive measures for individual patients: 10 at USB and 14 at UPK.^2^ Thus, such issues are slightly more frequent in the UPK ECs than in the USB ECs (58.3 and 41.7%, respectively). Most requests for such ECs come from adult psychiatry/UPK (58.3%), followed by requests from the medical division/USB (29.2%), and—with a clear distance—the surgical division/USB (8.3%). Physicians request EC more often (66.7%) than other professions, such as nurses (20.8%) or psychologists (8.3%) do. Patients or surrogates attend small minority of UPK- and USB-ECs only (4.2 and 12.5%, respectively). In almost half of the EC cases (45.8%), the written request for the EC did not specify—explicitly or implicitly^3^—“coercion” as the ethical issue in question beforehand. In these cases, the ethical issue of coercion is articulated during the EC for the first time, and its explicit articulation may be part of the clarification process.

### Patient Characteristics

The median age of the patients is 47.0 years, and all patients are adults (range 20–70 years). Female patients are more often discussed in ECs addressing coercive measures (70.8%) than in ECs in total (57.0%). Almost two thirds of the patients have a combination of somatic and psychiatric diseases (62.5%), one third suffering from psychiatric diseases only (33.3%), and one single patient in the sample has [according to International Classification of Diseases, 10th Revision (ICD-10)] just a somatic disease. Prognosis regarding the main medical issue of the patient is (according to caregivers) most often uncertain (33.3%) or poor (29.2%) but never terminal. However, good recovery is judged probable only in two patients. Patient decisional capacity is (according to caregivers) given in none of the ECs addressing coercive measures. In three fourths of the ECs, capacity is either impaired or not given (41.7 and 33.3%, respectively); in one fifth, it is unclear, whether it is given or not. Capacity is, thus, significantly more often compromised in ECs addressing coercive measures than in ECs in total (binary logistic regression test: OR 1.845, 95% CI, *p* = .015). An advance directive (AD), according to the caregivers, is available only for one patient (information on AD is only available in 41.7%). Demographic and clinical characteristics are shown in **Table 1**.

**Table 1:** Demographic and clinical characteristics of patient discussed in ethics consultation (EC).

Characteristic	Hospital		Total (*n* = 24)	% of total (*n* = 24)
	University Hospital Basel (USB) (*n* = 10)	Psychiatric Hospitals of the University Basel (UPK) (*n* = 14)		
Sex				
*Female*	7	10	17	70.8%
*Male*	3	4	7	29.2%
Age				
*Median age (year) (range)*	52.1 (28–69)	43.0 (20–70)	47.0 (20–70)	
Type of disease				
*Somatic disease*	1	0	1	4.1%
*Psychiatric disease*	1	7	8	33.3%
*Somatic and psychiatric disease*	8	7	15	62.5%
Decisional Capacity				
*Given*	0	0	0	0% (0%*)
*Impaired*	4	6	10	43.5% (41.7%*)
*Not given*	6	2	8	34.8% (33.3%*)
*Unclear*	0	5	5	21.7% (20.8%*)
*Not available*	0	1	1	4.2%
Prognosis				
*Good*	1	1	2	8.3% (9.5%*)
*Guarded*	2	2	4	16.7% (19.0%*)
*Poor*	3	4	7	29.2% (33.3%*)
*Terminal*	0	0	0	0% (0%*)
*Unclear*	2	6	8	33.3% (38.1%*)
*Not available*	2	1	3	12.5%
Advance directive (AD) available				
*Yes*	1	0	1	4.2% (10.0%*)
*No*	3	6	9	37.5% (90.0%*)
*Not available*	6	8	14	58.3%

*Including missing data.

### Coercive Measures

In slightly more than one third of all 24 ECs, the participants of the EC (including the ethics consultant) agreed on *applying* one or more coercive measures for the patient in question as the best course of action (37.5%). Coercive measures most often agreed upon were involuntary committal (25.0%), followed by compulsory treatment (20.8%). This equals the frequency of ECs in which the participants did agree on *not applying* such measures as best course of action. In one quarter of the ECs, the participants left it *open*, whether performing coercive measures can be recommended at this time, e.g. due to missing information or other available options to be tested first, with priority. In some cases, coercive measures (e.g. involuntary admission) had already been applied before the EC took place (41.7%)^4^.

Several types of coercive measures were discussed in the ECs: compulsory treatment (70.8%) such as compulsory pharmacological treatment, artificial nutrition, sedation, or diagnostics; involuntary committal (50.0%); and measures restricting liberty such as mechanical restraints or isolation (16.6%).

The spectrum of ethical reasoning as documented includes a variety of reasons pros and cons coercion that can be categorized according to the four principle approaches. All reasoning pros coercion (“pros”) considers reasons referring to beneficence, such as prevention of dying, protection of patient best interests (relating to health or quality of life), protection from patient self-harm, or averting risks to third parties. Reasons referring to respect for autonomy are considered in two thirds of all reasoning pros (66.7%). These reasons relate to the issue of the patient’s lacking decisional capacity, arguing that the patient’s wishes against treatment are not autonomous due to his/her (temporary) incapacity and/or that compulsory treatment may restore decisional capacity and, thus, support his/her autonomy. Some reasons pros refer to the principle of justice, e.g. that not to treat the patient coercively would contribute to an unjustified undertreatment or to ineffective use of resources (29.2%). Only in one case a reason pros is mentioned referring to non-maleficence: involuntary committal would terminate an existing disruptive “therapeutic relationship” and allow the patient to build up a new and better one (4.2%).

Reasoning cons coercion (“cons”) most often appeals to reasons referring to respect for autonomy, namely, that coercive treatment would inflict the right of the patient to decide about his/her own treatment (83.3%). In half of all reasoning cons, the principle of beneficence is referred to. It is argued that coercive measures would have a low probability of success or would not improve the patient’s situation regarding his/her health or quality of life. One third of the reasoning cons appeals to reasons related to non-maleficence, namely, that coercive measures would have high risks of aversive effects for the patient. Only one case refers to a reason cons about justice, i.e., that there are no institutions available to treat the patient effectively against his/her will (4.2%).

The characteristics related to coercion and reasoning about coercion are shown in **Table 2**.

**Table 2:** Type of coercion and reasoning.

Characteristics	Hospital		Total	% of total
	University Hospital Basel (USB)	Psychiatric Hospitals of the University Basel (UPK)		
Coercion as main issue in EC	*(n = 50)*	*(n = 50)*	*(n = 100)*	
*Yes*	10	14	24	–
*No*	40	36	76	–
Type of coercion addressed*	*(n = 10)*	*(n = 14)*	*(n = 24)*	*(n = 24)*
*Involuntary committal*	5	7	12	50.0%
*Compulsory treatment*	7	10	17	70.8%
*Measures restricting liberty*	2	2	4	16.6%
Type of coercion according to conclusion*				
*Involuntary committal*	3	3	6	25.0%
*Compulsory treatment*	2	3	5	20.8%
*Measures restricting liberty*	0	1	1	4.2%
Previous involuntary committal				
*Yes*	3	7	10	41.7%
*No*	7	7	14	58.3%
Request includes issue coercion				
*Yes, explicitly*	2	5	7	29.2%
*Yes, implicitly*	2	4	6	25.0%
*No*	6	5	11	45.8%
Reasons pro coercion*				
*Respect for autonomy*	6	10	16	66.7%
*Beneficence*	10	14	24	100%
*Non-maleficence*	0	1	1	4.2%
*Justice*	3	4	7	29.2%
Reasons con coercion*				
*Respect for autonomy*	6	14	20	83.3%
*Beneficence*	4	8	12	50.0%
*Non-maleficence*	3	5	8	33.3%
*Justice*	0	1	1	4.2%

*Multiple selection possible.

### Evaluation of EC—Outcome Criteria

All included ECs led, according to the documentation, to a consensus shared by the participants of the EC; this includes a newly formed explicit agreement. Characteristically, these agreements also cover procedural aspects such as who should try to convince the patient or other decision makers in order to still prevent the coercive measure. EC outcomes were, as far as reported, always implemented in practice afterward. EC meetings and written records are considered helpful by the requestors/feedback respondents in all cases. Outcome criteria are shown in **Table 3**. However, unavailable data are more frequent than optimal in this sample.

**Table 3:** Evaluation of EC—outcome criteria.

Characteristic	Hospital		Total (*n* = 24)	% of total (*n* = 24)
	University Hospital Basel (USB) (*n* = 10)	Psychiatric Hospitals of the University Basel (UPK) (*n* = 14)		
Consensus				
*Yes*	9	13	22	100%(91.7%*)
*No*	0	0	0	0% (0%*)
*Not available*	1	1	2	8.3%
Novelty of the consensus				
*Yes*	8	13	21	100% (87.5%*)
*No*	0	0	0	0% (0%*)
*Not available*	2	1	3	12.5%
Results implemented				
*Yes*	3	6	9	100% (37.5%*)
*No*	0	0	0	0% (0%*)
*Not available*	7	8	15	62.5%
EC/written records found helpful by requestor				
*Yes*	3	10	13	100% (54.2%*)
*No*	0	0	0	0% (0%*)
*Not available*	7	4	11	45.8%

*Including missing data.

## Discussion

Clinical staff appears more sensitive to perceiving ethical uncertainty or conflict than being prepared to articulate a focus of ethical concern in precise terminology, especially regarding coercion. EC meetings have, thus, inter alia the role to identify or “diagnose” the ethics focus of the problem(s) presented in EC and to bring forward specific solution(s) according to the concept of ethics facilitation (22).

### Patient Characteristics

Coercive measures are not only a matter of reflection in EC concerning the psychiatric patients in the sample (14 out of 24); they are also discussed in some cases of the somatic hospital (10 out of 24). Even there, patients may show complex conditions connected with psychiatric symptoms, especially loss of capacity, sometimes in connection with lacking insight and adherence contributing to deterioration of physical health.

Of the few studies quantifying ethical issues discussed in EC, most do not report figures relating to coercion (25–29). Some of them, however, report how frequently patient “autonomy” or “refusal” was the central topic in EC. Unfortunately, this may or may not include the discussion of coercion as long as it is not mentioned or excluded explicitly in the used category system. Without any standardized categorization of EC content such as coercion, comparative EC research will, thus, remain impossible.

Patient decisional capacity proved to be a key component of ethical reasoning, especially in relation to the duty to prevent harm. Alone, it is not a sufficient reason to justify coercion. According to the data, capacity had been qualified uncertain by the clinicians in one-fifth stimulating questions about the procedure and quality of assessment in practice. As capacity is significantly more often compromised in ECs addressing coercive measures than in ECs in total, it has to be acknowledged that this patient characteristic deserves the utmost attention and carefulness. As far as the professional judgment of capacity is made in a less than systematic way, the rationality or even ethicality of decision making on coercion may be impaired. This problem has recently been acknowledged by the Swiss Academy of Medical Sciences, issuing its new guideline on capacity by offering recommendations and protocols for capacity assessment that appeared after the EC cases took place (2018).

Only in 1 out of 24 cases was an AD known of or available. This is an alarming result: given the fact that loss of capacity repeatedly occurs after recurrent clinical signals, it is wondered why more patients had not been forewarned and prepared timely, e.g. by the clinicians involved. Switzerland adopted the Child and Adult Protection Law (as part of the SCC Revision) in 2013 supporting patient ADs that are legally binding when applicable. The USB provides internal AD tools; the staff is obliged to ascertain on admission any existing AD. The UPK support the use of Ads, and psychiatry-specific treatment agreements and internal tools are also available for staff education and patient counseling. Advanced decision making of this kind would be of great value to both patient and caregivers. In the EC approach practiced here, the role of EC and session chair is included, where appropriate, especially when incapacitation seems a forthcoming threat to the patient, raising the question whether an AD should be suggested to the patient—always in combination with counseling. Also, CES is offered in this regard for cases where need be.

### Coercive Measures

Restriction of privacy or freedom of communication, detention of persons admitted voluntarily, or physical coercion (holding) were not discussed in these cases, although permissible under specific circumstances according to Swiss law. On the whole, the documented outcomes suggest the hypothesis that using EC does not predetermine whether or not coercion will actually be applied in a case. Such an unprejudiced attitude may be appreciated as indicating open-mindedness. It may also be challenged, however, in the light of guidelines and policies that are dedicated to actually reducing the frequency of coercion in patient care. The S3-Guideline of the German Deutsche Gesellschaft für Psychiatrie und Psychotherapie, Psychosomatik und Nervenheilkunde (DGPPN; German Association for Psychiatry, Psychotherapy and Psychosomatics) on the prevention of coercion: “prevention and treatment of aggressive behavior in adults” is also followed in Switzerland, too. As its title reveals, its goal is the abandonment of coercion altogether (8), and it does not address indications for coercive measures in nonaggressive patients with intentions such as, e.g. life-sustainment. In contrast, the Swiss guideline “coercive measures in medicine” does not run under a prohibitive title similar to DGPPN (12). It states in its preamble:

“The guidelines are designed to promote and maintain awareness of the fact that coercive measures of any kind—even if they comply with all the relevant procedural requirements—represent a serious infringement of fundamental personal rights and thus require ethical justification in each case. (…) In all cases, careful ethical reflection is just as indispensable as rigorous compliance with legal provisions and applicable guidelines” (p. 5) (12).

A wider range of phenomena and areas of application is indeed covered: Patients with somatic as well as mental disorders, children and adolescents, patients in long-term or in domiciliary care, and finally, patients undergoing execution of sentences and (forensic) measures. It is, thus, more applicable to the EC context. Even though the Swiss guideline does not request that coercion be foregone completely, it insists that it should be ethically reflected—as is the case in EC: Is the practice of EC in Basel to be criticized for not fighting strongly enough against coercion? Would we expect EC cases to go without coercion as the consultation process mobilizes potential for other, better problem solutions?

As a matter of fact, the UPK are ranging relatively low in applying coercive measures in light of the Swiss report on psychiatric institutions (30). As the ANQ statistics show, approximately 3,000 cases (4.3% of all cases) fall under the category “measures restricting liberty” (German: “Freiheits-beschränkende Massnahmen”), which includes compulsory treatment, but excludes involuntary committal. While numbers such as these would exceed the existing availability of case-based ethics support, it does make perfect sense to consider implementing prospective EC before specific measures such as coercive treatment (medication) are decided for patients under certain conditions, especially loss of decisional capacity. Moreover, this could and should be complemented by retrospective ethical case discussions on a more regular basis as was put to the test in the context of opening doors (24). Retrospective ethical case discussions could be practiced institution-wise and within the framework of model projects accompanied by evaluation. Practiced in larger rounds, they could cover considerable numbers of interdisciplinary staff by supporting them to anticipate and master ethical challenges. Preventing coercion on a large scale requires the implementation of educational strategies and efforts to professionalize prevention and de-escalation of patient aggression, additionally [see Ref. (8)]. Both USB and UPK have engaged in related activities (USB guideline on violence in patients and relatives; USB minimal standard of restraint). However, in the UPK, such guidelines exist, for internal use only.

### Reasons and Ethical Reasoning

Ethical reasoning about the competing options is crucial for an unprejudiced decision complying with the normative framework and for building a robust consensus. Also, in our EC practice, core requirements of the DGPPN, as well as the SAMS guidelines such as the proportionality and the priority on using the minimally invasive/coercive measures, are explicitly followed. However, the application of coercion may, in the individual case, save life rather than accept premature dying, terminate reversible suffering rather than tolerate severe symptoms, and help to rebuild patient autonomy, i.e., capacity to engage in advance care planning, instead of watching a patient’s deterioration of personality. The recovery of patient autonomy is, in fact, one of our preferred outcomes.

### Evaluation of EC—Outcome Criteria

In general, requestor feedback on EC, where available, is more than appreciative. Specifically, the outcome that the consensus built in EC was new compared with the situation prior to the EC meeting, which was a crucial one: It corroborates the idea that EC is not about holding an understanding conversation, albeit being of an educational or psycho-hygienic value; it does not function like psychological supervision either (31). Rather, it offers specific components and concrete steps to methodically analyze and solve ethical problems in a clinical context (20, 22, 23). Two more outcome criteria are important in the evaluation: the implementation of EC results and the helpfulness (of both interactive sessions and written records) as experienced by the requestors. Both criteria are rated very high, but serve to only roughly estimate the value of the respective EC. Further elaboration of the concepts is, in our view, reserved to in-depth qualitative single case studies. Also, the return rate in this sample was with 54.2% rather moderate; we have, however, succeeded since in obtaining more regularly and frequently, feedback from requestors by using active reminders.

### Limitations of the Study

No empirically validated judgment is provided about the quantitative proportion of cases including coercive measures in the two clinical settings in general and the respective EC cases. This kind of analysis has to await more comprehensive national epidemiological data on coercion at large. However, as the kind of study reported here is rather innovative in using a comprehensive database on EC cases, the results are certainly relevant to encouraging further investigations on EC in relation to coercion.

## Conclusions

Coercive measures and their ethical legitimation are a matter not only in psychiatric EC but also in the acute somatic context.

Patient decisional capacity is more often compromised in ECs addressing coercive measures than in ECs in total; this relation requires further study. It is suggested making more efforts toward early and professional assessment of patient capacity.

Likewise, missed opportunities to forewarn and prepare patients timely, in combination with counseling them on an advance directive, should be investigated more extensively to pave the way toward improvements.

It is put to discussion whether EC should be recommended for standard use whenever coercion is an issue in patient care—ideally before it may be applied, or, otherwise in retrospect for quality development.

In order to allow for comparative EC research on a systematic basis, shared standardized categorization of EC content, e.g. regarding coercion, is indispensable.

## Ethics Statement

Considering the approach of our case series, the Ethical Committee for North-western and Central Switzerland (EKNZ) granted an exemption from requiring ethics approval and consent to participate. Waiver was issued on 26.03.2019 by Nieke Jones, head of the scientific secretariat EKNZ.

## Author Contributions

EM and SR-T designed the study and drafted the manuscript. EM and JS carried out the case analyses. JS, CW, and SR-T contributed to the analysis and interpretation of data and worked on the text and references. SR-T supervised the study and revised the manuscript. MP read and approved of the manuscript. EM, JS, CW, and SR-T read, edited, and approved of the final manuscript; they also took care of the revision.

## Conflict of Interest Statement

The authors declare that the research was conducted in the absence of any commercial or financial relationships that could be construed as a potential conflict of interest.

## Footnotes

1 According to Beauchamp and Childress, the principle of non-maleficence refers only to the duty not to inflict evil or harm. The duty to prevent or remove harm, e.g. the concept of harm reduction, refers to the principle of beneficence.

2 The ethical issues of all 100 EC cases are reported in Reiter-Theil and Sch&amp;uuml;rmann (13).

3 A request identifies coercion implicitly, if it states measures that are against the will of the patient.

4 This did not affect the numbers of EC that resulted in agreement for coercive measures.
